# Supplementary material for: Preparation and Characterization of Flavored Sauces from Chinese Mitten Crab Processing By-Products
Source: Foods. 2022 Dec 22;12(1):51. doi: 10.3390/foods12010051 (PMC9818834; doi:10.3390/foods12010051)
Supplement: Supplementary file 1 [file foods-12-00051-s001.zip › Supplementary Information1.doc]

Supplementary Information

**Preparation and characterization of sauces from Chinese mitten crab processing by-products**

ZHOU Yan ^a^, ZHAO Tianyu ^a^, WANG Zhenmeng ^a^, XV Jiachao ^a^, SUN Jianan^a,^ ^*^, MAO Xiangzhao ^a,b,*^

^a^ College of Food Science and Engineering, Ocean University of China, Qingdao 266003, China

^b^ Laboratory for Marine Drugs and Bioproducts, Qingdao National Laboratory for Marine Science and Technology, Qingdao 266237, China

*Corresponding author: Jianan Sun, Xiangzhao Mao

Address: College of Food Science and Engineering, Ocean University of China, No. 5 Yushan Road, Qingdao 266003, China

Tel.: +86-532-82031360;

Fax: +86-532-82032272

E-mail:[sunjianan@ouc.edu.cn](mailto:sunjianan@ouc.edu.cn), [xzhmao@ouc.edu.cn](mailto:xzhmao@ouc.edu.cn)

**1. Supplementary tables**

Table 1 Sensors description and other related information in E-nose

| Sensors | Description | Representative compounds | Response threshold (ml/m^3^) |
| --- | --- | --- | --- |
| R（1） | Aromatic | Toluene | 10 |
| R（2） | Broad range | NO_2_ | 1 |
| R（3） | Aromatic | benzene | 10 |
| R（4） | Hydrogen | H_2_ | 100 |
| R（5） | Arom-aliph | Propane | 1 |
| R（6） | Broad-methane | CH_4_ | 100 |
| R（7） | Sulphur-organic | H_2_S | 1 |
| R（8） | Broad-alcohol | CO | 100 |
| R（9） | Sulph-chlor | H_2_S | 1 |
| R（10） | Methane-aliph | CH_4_ | 10 |

Table 2 Basic components of CLs and CBs from Chinese mitten crab (%)

|  | Moisture | Ash* | Crude protein* | Crude lipid* | Crude chitin* |
| --- | --- | --- | --- | --- | --- |
| CL | 50.60±1.16 | 46.52±0.55 | 42.37±0.18 | 1.26±0.34 | 8.01±0.75 |
| CB | 58.28±0.27 | 41.30±0.21 | 22.43±2.09 | 5.30±0.18 | 19.91±0.92 |

Data are means ± SD;* Dry matter; the crab legs (CLs) and crab bodies (CBs)

Fig. 1 E-nose patterns: (a) ten sensors response to volatile components from HO of the CLs (b) ten sensors response to volatile components from EHS of the CLs (c) ten sensors response to volatile components from MRS of the CLs (d) ten sensors response to volatile components from HO of the CBs (e) ten sensors response to volatile components from EHS of the CBs (f) ten sensors response to volatile components from MRS of the CBs
